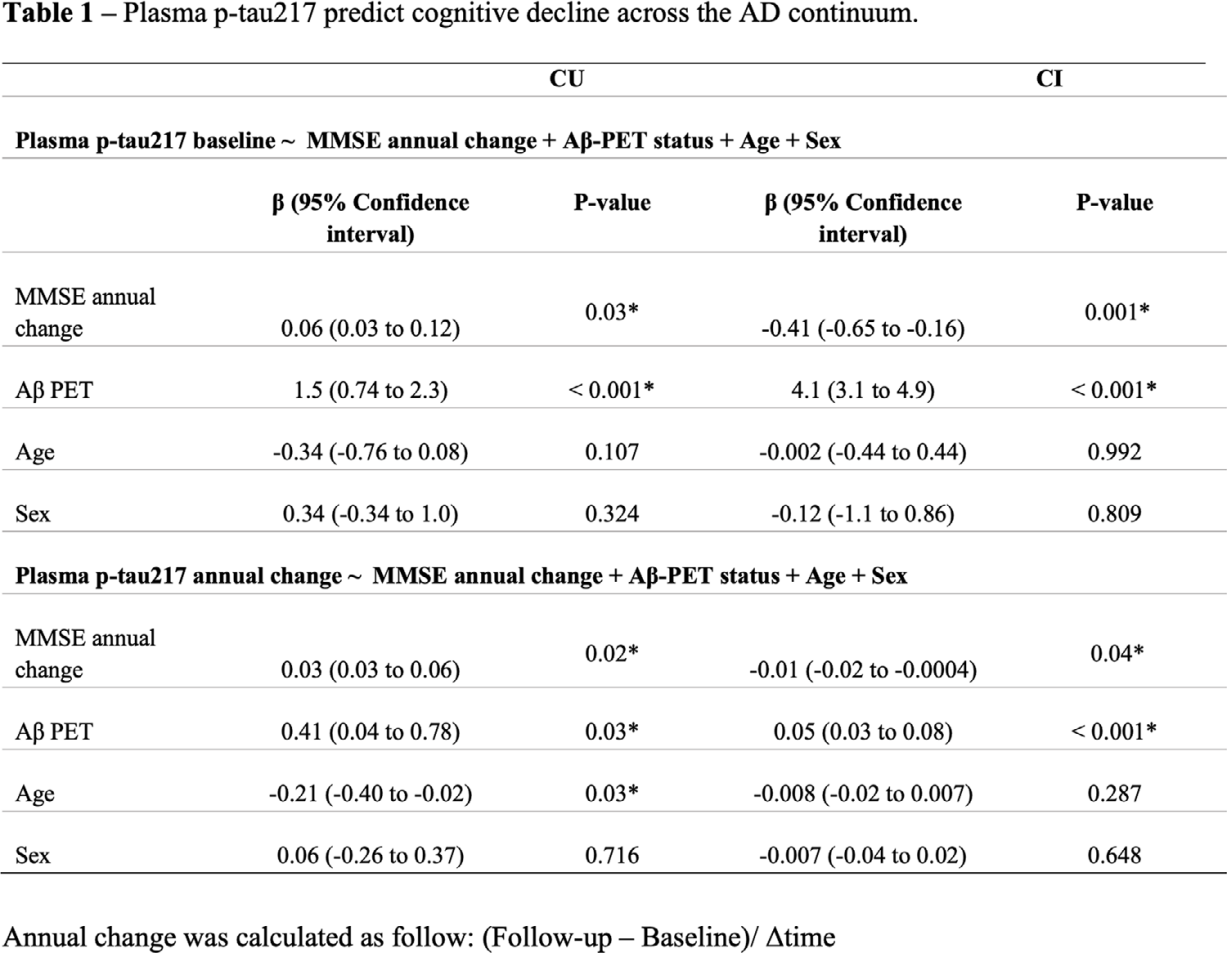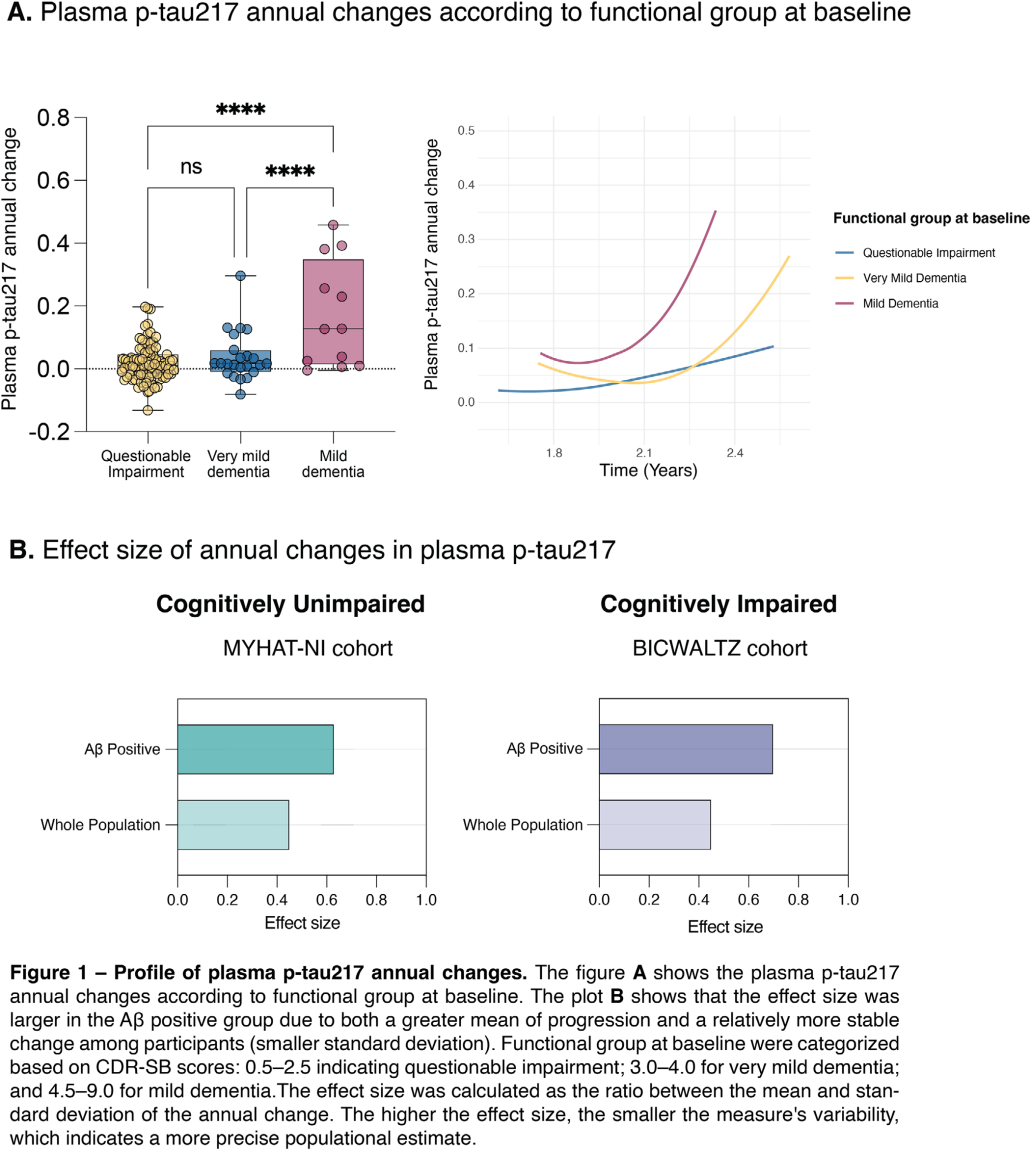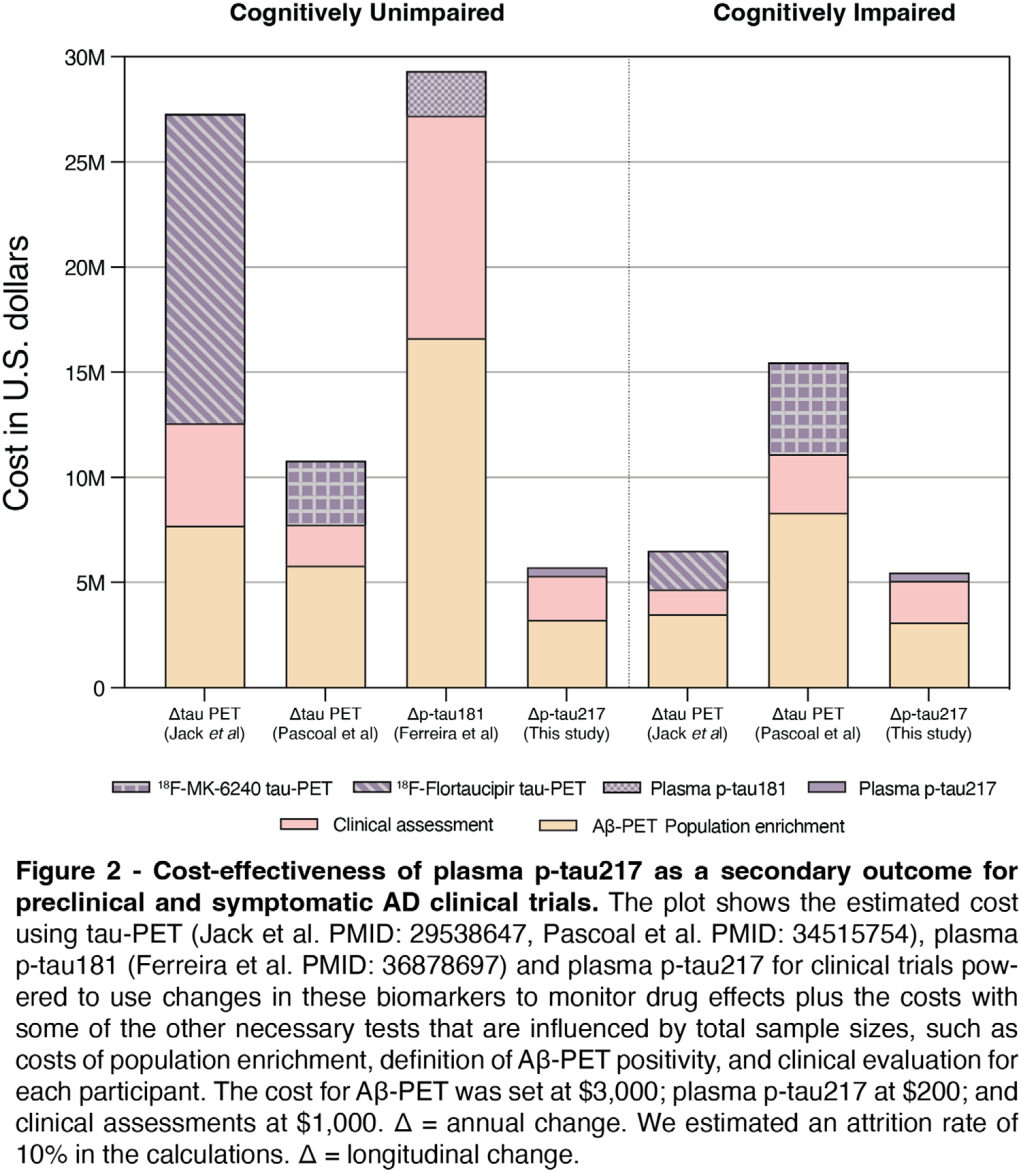# Utility of plasma p‐tau217 as a secondary outcome for clinical trials across AD continuum

**DOI:** 10.1002/alz.094020

**Published:** 2025-01-09

**Authors:** Pamela C.L. Ferreira, Bruna Bellaver, Guilherme Povala, Cristiano Schaffer Aguzzoli, João Pedro Ferrari‐Souza, Douglas Teixeira Leffa, Firoza Z Lussier, Marina Scop Madeiros, Hussein Zalzale, Francieli Rohden, Carolina Soares, Sarah Abbas, Guilherme Bauer‐Negrini, Helmet T. Karim, Chang Hyung Hong, Hyun Woong Roh, Dana Tudorascu, Thomas K Karikari, Beth E. Snitz, Sang Joon Son, Tharick A. Pascoal

**Affiliations:** ^1^ Department of Psychiatry, University of Pittsburgh School of Medicine, Pittsburgh, PA USA; ^2^ Universidade Federal do Rio Grande do Sul, Porto Alegre, Rio Grande do Sul Brazil; ^3^ University of Pittsburgh, Pittsburgh, PA USA; ^4^ Brain Institute of Rio Grande do Sul, Porto Alegre, RS Brazil; ^5^ Ajou University School of Medicine, Suwon Korea, Republic of (South); ^6^ Ajou University School of Medicine, Suwon, Gyeonggido Korea, Republic of (South); ^7^ University of Gothenburg, Mölndal Sweden

## Abstract

**Background:**

Blood‐based biomarkers offer a cost‐effective and simple alternative for clinical use in the context of Alzheimer’s disease (AD). It has already been shown that plasma phosphorylated tau at threonine 217 (p‐tau217) is associated with amyloid (Aß), neurofibrillary tau tangles, and cognitive decline. Longitudinal studies confirmed its ability to track AD progression. Therefore, the aim of this study is to evaluate the potential utility of changes in plasma p‐tau217 as secondary outcome in clinical trials across the AD spectrum.

**Method:**

We included 189 individuals with available plasma p‐tau217 measurements at two time points (24 months follow‐up) across two cohorts:[MYHAT‐NI, population‐based(USA): 51 Cognitively Unimpaired(CU); BICWALZS, memory clinic(South Korea): 135 Cognitively Impaired(CI) individuals], all with baseline Aß‐PET. Plasma p‐tau217 was quantified using the ALZpath assay. We used linear regression to investigate p‐tau217 association with cognitive decline. Effect size was determined by biomarker mean change divided by the standard deviation. We also estimated the sample size required to detect a 25% drug effect on p‐tau217 reduction with 80% power at a significance level of 0.05.

**Result:**

Baseline and longitudinal changes in p‐tau217 were significantly associated with cognitive decline independently of Aß status in both CU and CI individuals (Table 1). Functionally, individuals classified with mild dementia(CDR‐SB>4.5) showed greater and pronounced annual changes in p‐tau217 compared to the other groups (Figure 1A). Notably, Aß+ individuals presented a higher effect size for p‐tau217 changes in both CU(0.79) and CI(0.74) groups, compared to the whole population (Figure 2B). The sample size required for a clinical trial including of Aß+ individuals were 1,276 for CU and 940 for the CI. Cost analysis revealed that in trials recruiting CU Aß+ individuals targeting tau pathology using p‐tau217 as a secondary outcome was 1.5, 3.7, and 3.9 times more cost‐effective than 18F‐MK‐6240 tau‐PET, 18F‐Flortaucipir tau‐PET, and p‐tau181, respectively (Figure 2). Similarly, in trials with CI Aß+ participants, p‐tau217 was 1.5 times less expensive than [18F]‐MK‐6240 tau‐PET, and 3.7 times less than [18F]‐Flortaucipir tau‐PET.

**Conclusion:**

Our results demonstrated that changes in plasma p‐tau217 is associated with cognitive decline across the AD continuum. Furthermore, p‐tau217 shows promise as a secondary outcome in AD clinical trials.